# Supplementary material for: Comparative multiomics analysis of cell physiological state after culture in a basket bioreactor
Source: Sci Rep. 2022 Nov 23;12:20161. doi: 10.1038/s41598-022-24687-4 (PMC9686226; doi:10.1038/s41598-022-24687-4)
Supplement: Supplementary file 1 — Supplementary Information 1. [file 41598_2022_24687_MOESM1_ESM.zip › raw data/Proteomic raw data/3.ProtExprQuantification/cv_analysis.pdf]

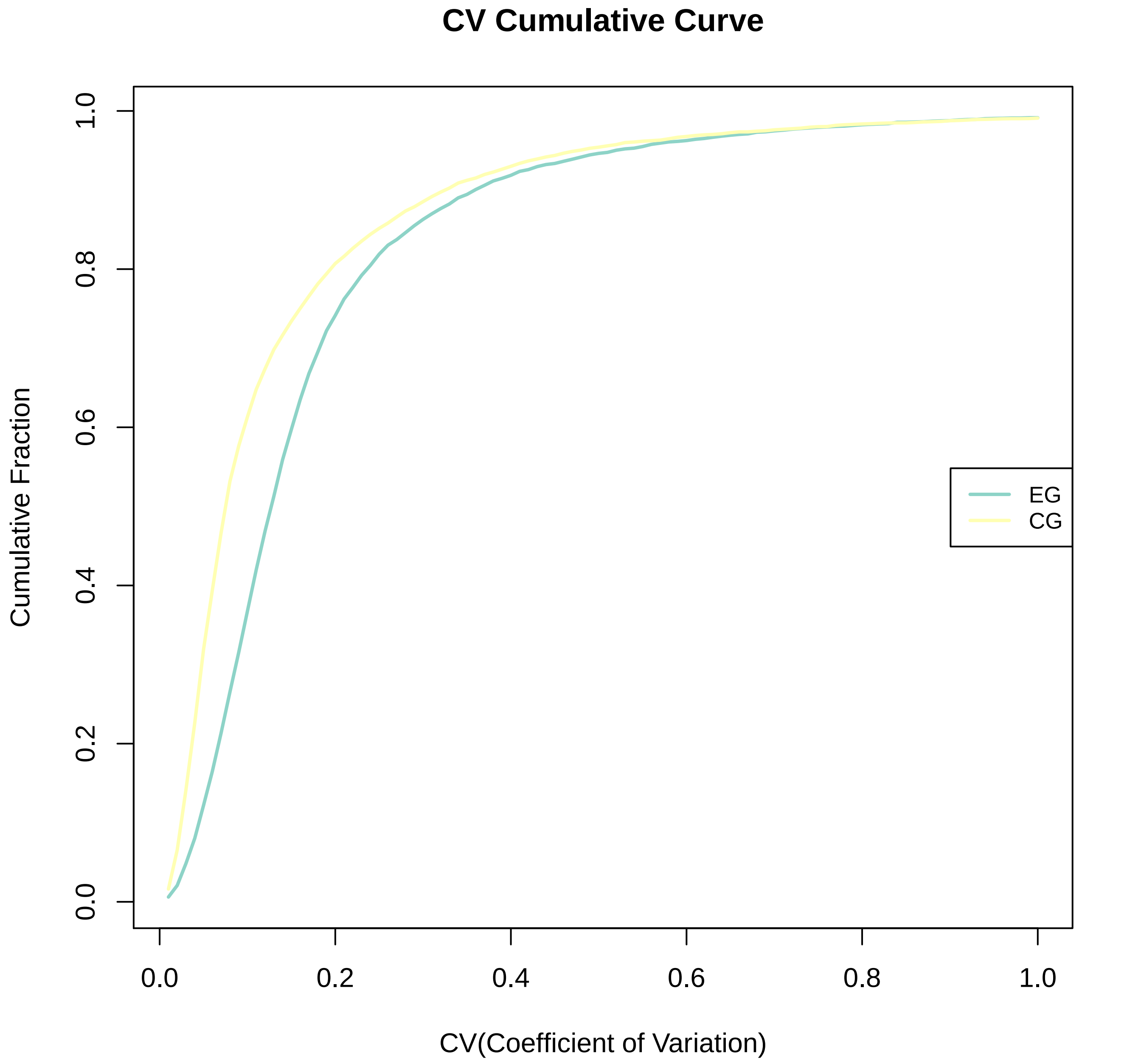

CV Cumulative Curve
Cumulative Fraction
CV(Coefficient of Variation)
0.0
0.2
0.4
0.6
0.8
1.0
EG
CG
